# Geographic Wormhole Detection in Wireless Sensor Networks

**DOI:** 10.1371/journal.pone.0115324

**Published:** 2015-01-20

**Authors:** Mehdi Sookhak, Adnan Akhundzada, Alireza Sookhak, Mohammadreza Eslaminejad, Abdullah Gani, Muhammad Khurram Khan, Xiong Li, Xiaomin Wang

**Affiliations:** 1 Center for Mobile Cloud Computing (C4MCC), University of Malaya, Kuala Lumpur, Malaysia; 2 Fiber Optics Communication Networks Project Manager, Fars Regional Electric Co., Shiraz, Iran; 3 Faculty of Computing and Information System, Universiti Teknologi Malaysia, Johor, Malaysia; 4 Center of Excellence in Information Assurance (CoEIA), King Saud University, Riyadh, Saudi Arabia; 5 School of Computer Science and Engineering, Hunan University of Science and Technology, Hunan 411201, Xiangtan, China; 6 School of Information Science & Technology, Southwest Jiaotong University, Chengdu, China; Tianjin University of Technology, CHINA

## Abstract

Wireless sensor networks (WSNs) are ubiquitous and pervasive, and therefore; highly susceptible to a number of security attacks. Denial of Service (DoS) attack is considered the most dominant and a major threat to WSNs. Moreover, the wormhole attack represents one of the potential forms of the Denial of Service (DoS) attack. Besides, crafting the wormhole attack is comparatively simple; though, its detection is nontrivial. On the contrary, the extant wormhole defense methods need both specialized hardware and strong assumptions to defend against static and dynamic wormhole attack. The ensuing paper introduces a novel scheme to detect wormhole attacks in a geographic routing protocol (DWGRP). The main contribution of this paper is to detect malicious nodes and select the best and the most reliable neighbors based on pairwise key pre-distribution technique and the beacon packet. Moreover, this novel technique is not subject to any specific assumption, requirement, or specialized hardware, such as a precise synchronized clock. The proposed detection method is validated by comparisons with several related techniques in the literature, such as Received Signal Strength (RSS), Authentication of Nodes Scheme (ANS), Wormhole Detection uses Hound Packet (WHOP), and Wormhole Detection with Neighborhood Information (WDI) using the NS-2 simulator. The analysis of the simulations shows promising results with low False Detection Rate (FDR) in the geographic routing protocols.

## Introduction

Security of wireless sensor networks (WSNs) has gained considerable interest more recently. Mainly because the sensory nodes, having limited computational and communicational resources, providing a secure routing protocol is performing a complex task in WSNs [1, 2].

Routing in WSNs is challenging because of unique characteristics that make WSNs different from other wireless networks, such as Mobile Ad Hoc Networks (MANET). Such characteristics consist of (a) Traditional IP-based methods for WSNs are inapplicable because of the deployment of the large number of sensor nodes in the network. (b) The majority of applications of sensor networks transmit the data to a specific base station. (c) Sensor nodes require a particular resource management due to the fact that the battery, storage, and processing capabilities of sensors are limited. (d) In contrast to traditional wireless networks, almost all nodes in WNSs are immobile after deployment or have a very low mobility. (e) The sensor nodes in WSNs require a particular hardware (e.g., GPS) to find the position of the other nodes due to the location-based data collection. (f) To augment the energy and bandwidth utilization of the routing protocols in WSNs, the data redundancy during data collection has to be reduced. Despite the fact that considerable progress has been made in the past few years pertaining to advancement in WSNs, providing a secured routing protocol has received more attention from the researchers [3–7]. There are several attacks that threaten the security of the routing protocols in WSNs that cause different problems, such as changing the routing protocol and threatening confidentiality, availability, and integrity of the transmitted packets. The WSN security attacks include selective forwarding, sinkhole, Sybil, wormholes, hello flood, and acknowledgement spoofing attacks [8–12]. However, the wormhole attack is more harmful than the others, because it does not need to compromise a sensory node within the network. Moreover, the wormhole attack is able to easily cause other types of attacks, such as Sybil attack. Furthermore, using a cryptographic technique cannot prevent wormhole attacks [13–16].

Significant amount of work has recently been carried out to prevent the wormhole attacks in wireless ad hoc networks. Such methods typically detect the attacks on the basis of indications that are generated by the wormholes. However, most of the existing methods require either particular hardware devices e.g., GPS [13, 17], directional antennas [18], special radio transceiver modules [19], or strong assumptions on the networks e.g., precise synchronized clock [13], guard nodes [20], unit disk communication models [21], or safe and attack-free environments [22]. Employing such requirements and assumptions restrict the application of the methods in geographical routing protocols that include a huge number of resource-constrained sensor nodes.

This paper presents a unique method to detect the dynamic and static wormhole attacks in geographic routing protocols (DWGRP). The main contribution of this paper is to create a new type of pairwise key predistribution [23] based on the beacon packets in order to detect the malicious nodes more efficiently. The proposed method is able to detect malicious nodes and select the best and most reliable neighbor without any specific assumptions and is not subject to any requirements for extra hardware devices. The simulation results demonstrate the effectiveness of the proposed method and indicate that the probability of wormhole detection in the DWGRP method is considerably higher than the related techniques.

### 0.1 Geographical Routing Protocols

Location-based routing protocols are an important group of protocols in WSNs in which position information is used to route data towards the desired regions (sinkhole). Location-based routing, is also known as position-based, directional, geographic, or geometric routing [24]. This section briefly reviews the geographic routing protocols.

**Figure 1:**
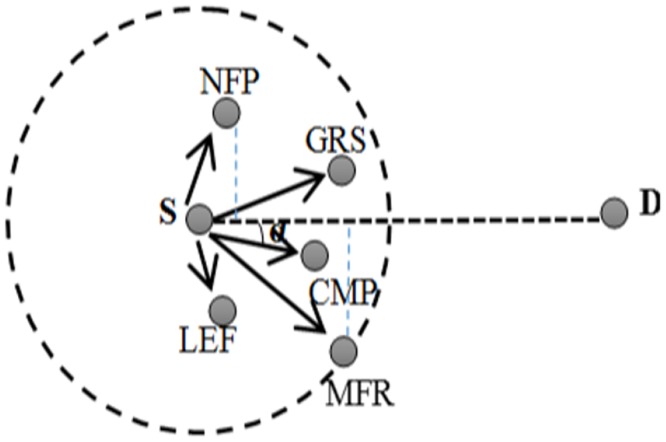
Forwarding approaches in geographic routing protocols.

The geographic routing protocols are classified into five groups, based on how the next hop is chosen. The Greedy Routing Scheme (GRS) is the first group of geographic routing protocol in which each node selects the best node among the neighbors that is closest to the destination. GPSR is an example algorithm falls in this category in which a packet should be forwarded hop by hop based on GRS and available local information, which is actually gathered by the Global Positioning System (GPS) until it meet a void area. In this way, the received message must be passed to the first neighbor counterclockwise about itself [25]. The next group of the geographic routing protocols is called Most-Forward-within-R strategy (MFR). In MFR, the packet is sent to the most forward node to destination among the neighbors of the sender based on the transmission range (R). The third approach is the Nearest-Forward-Progress scheme (NFP) in which the nearest neighbor to the transmitter is chosen to send data. The compass routing scheme (CMP) is the fourth method among the geographical routing protocols. In this scheme, the neighbor that has a minimum angle to the imaginary line between the source and destination is selected as the next hop. Low-energy forward scheme (LEF) selects a neighbor that requires a minimum energy to transmit packets. However, among these geographic routing protocols, the GRS is more popular and more applicable than the other methods due to the rate of delay and energy of this method [26, 27]. Fig. 1 illustrates how the next node will be selected in the different type of forwarding approaches to transfer packet from source (S) to destination (D) node.

### 0.2 Complex Network

Complex network is theoretically defined as a graph with non-trivial topological, function and dynamical features of many real networks, which does not exist in a simple network such as lattices, random graphs, degree distribution, a high clustering coefficient, assortativity or disassortativity [28]. WSNs can be considered as a specific type of complex networks, because it includes large-scale distributed sensor nodes in which the sensor nodes have the capability to communicate with the sensor nodes in the predefined region.

Li et al. [29] presented a novel local-world model of WSN that consists of two types of nodes such as sensor node and sink node. The sensor nodes are responsible for gathering data information from the environment and transferring data to the sink nodes. The received data are finally sent to gateway through other sink nodes. The proposed model is able to balance the energy consumption by minimizing the connectivity of sink nodes on the basis of the energy of each sensor node. The authors also identified that the degree distribution can be described as an integral in relation to proportion of sink nodes and energy distribution by using mean-field theory [30].

The theory of complex network encounters new challenges due to the requirement of understanding appropriately the dynamical characterization of real systems, especially when the systems consist of two or more interconnected networks [31]. As a result, to understand the complicated changeability of real complex systems that include various time scales and structural patterns, it requires defining a new concept as a multiplex network. The multiplex network refers to a multilevel system in which each layer has particular and unique characteristics and the layers are connected by a richer structure of interactions. The interconnection between layers in such multilevel graph-based structure shows how the nodes of different levels are connected and influence each other. This type of graphs can be used to analyse numerous biological systems and many social networks, such as Twitter and Facebook [32].

Since the epidemics are able to spread across the multiplex network, extensive studies have been carried out to analyse dynamical epidemics in recent years. However, most of the existing studies only focused on epidemics spread over single transmission route. Zhao et al. [33] was the first to analyse multiple routes transmitted epidemic on the multiplex network and propose a two routes transmitted epidemic spreading on two network layers. This method is adopted the Susceptible Infected Removed (SIR) model [34] in which each node includes three compartments, such as susceptible (disposed to be infected), infectious (already infected), and recovered from disease. The authors also accurately calculated outbreak size of the epidemic and the epidemic threshold of the multiplex network. Moreover, they suggested two measures for determining the level of inter-similarity of two layers, such as average similarity of neighbors (ASN) and degree degree correlation (DDC). The ANS is used to evaluate the average of similarity of nodes in different layers while the DDC indicates the correlation of node’s degree in different layers.

One of the important application of the complex network is to analyze the traffic system of metropolises [35]. To achieve this goal, the researchers proposed multi-layered real-world systems as an interconnected network in which various social behavior roles are assigned to various layers. For example, in city traffic system, two interdependent networks need to be designed with two types of link, such as connectivity and dependency [36]. Recently, the researchers have found that the percolation and cascade failures properties in dynamic complex systems are affected by the topology of each interdependent layer. However, most of the existing models are unable to describe the complicated cascade failures of real complex traffic systems in which various attacking rules and load patterns exist simultaneously.

In [37], Su et al. considered this problem and designed a flow redistribution model for cascading failures on the basis of redistribution of traffic flow in two different types of interrelated networks, such as dependent network (subway network) and connected network (bus network). The authors exposed that there are non-equilibrium phase transitions at the point of low network capacity in the city traffic system. By using this characteristic, they explained why a small increase in the number of buses during rush hours has an impalpable effect on the traffic congestion. Moreover, they uncover that removing a node of the bus network randomly can cause a traffic jam. Nevertheless, when the damage is inconspicuous, this type of traffic jam can be released by increasing the number of buses.

Complex network also can be used to model the infectious diseases. In [38], Xia et al. explored the effects of delayed recovery and non-uniform transmission on the propagation of diseases on structured populations. They found that rescheduling the transition from the infectious to the recovered states resulted in diminishing the epidemic threshold. Sanz et al. [39] designed a comprehensive framework for explaining the spreading dynamics of two concurrent diseases. The authors also represented the epidemic thresholds of the two diseases and computed the temporal evolution to characterize the unfolding dynamics.

Our work is entirely based on simple classical networks, which includes a single layer. Considering it for multiplex networks will certainly have great contribution to the research community. Keeping in view, the complexity of multiplex networks; an extension of our work need to be redesigned and are considered in our future plans.

### 0.3 Wormhole Attack

**Figure 2:**
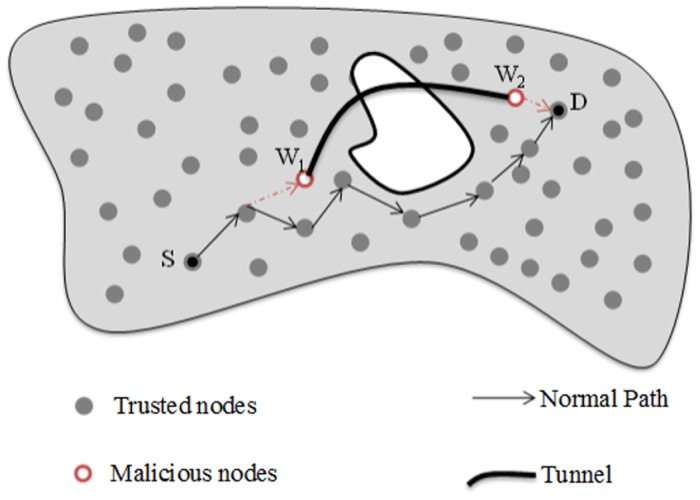
The wormhole attack.

Wormhole attack usually occurs by connecting at least two malicious nodes via an out-of-band connection that is called tunnel. The first malicious node eavesdrops or receives packets in one area and then tunnels the packets to the next malicious node that is placed at another point of the network. The tunnel is created either by using direct wired link or by using a long-range directional wireless link [40, 41]. Fig. 2 depicts the following scenario, since the source node (*S*) directs packets to the destination node through the normal path, however, in the case of a wormhole attack; the packets are actually eavesdropped by the first malicious node (*W*1) and then tunneled to second malicious node (*W*2). Consequently, *W*2 pass on the packets to the terminus node (*D*). The tunneled packets arrive earlier than the packets through normal path. Therefore, the remaining packets that follow the normal path will be dropped by the destination node.

**Figure 3:**
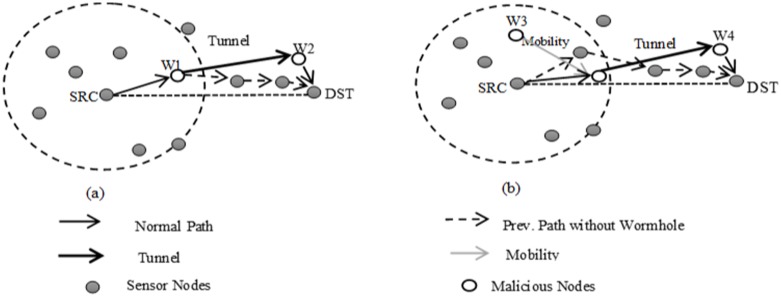
Wormhole attack in geographical routing protocols, (a) static wormhole, (b) dynamic wormhole.

The wormhole attacks can be categorized into two groups based on the type of malicious nodes, such as static and dynamic wormhole. The static wormhole attack occurs when the malicious nodes are statically located along the path to the destination. For example, the wormhole attack that is created by the malicious nodes *W*1 and *W*2 in Fig. 3.(a) is static. In the dynamic wormhole attack, initial deployments of the malicious nodes are not placed within the normal path to the destination. However, the malicious nodes are able to obtain the routing information by overhearing and processing the data packets. Afterwards, such nodes move toward the specific path for receiving the message and create the dynamic wormhole. As a result, identification of the dynamic wormhole is more difficult than the static type [40]. Fig. 3.b illustrates the dynamic wormhole attack.

The remaining paper is organized as follows. Section 2 presents a new classification technique of the wormhole detection methods to highlight the advantages and the disadvantages of the related works. Section 3 describes proposed wormhole detection method. The performance analysis of the proposed wormhole detection method and the comparison with other similar methods is presented in Section 4. Finally, the concluding remarks are provided in Section 5.

## 1 Related Works

**Figure 4:**
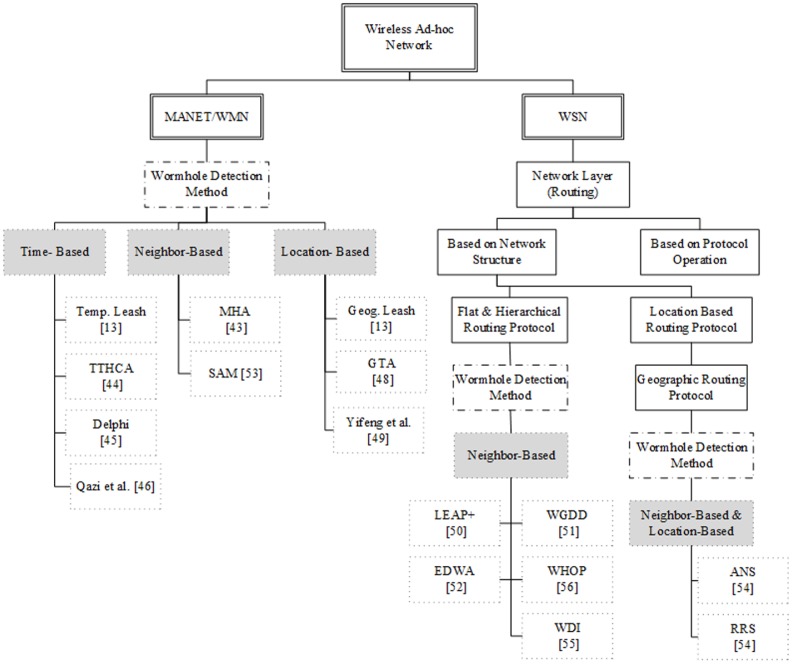
Taxonomy of wormhole detection methods.

Fig. 4 presents the new classification of wormhole detection methods on various routing protocols on the basis of the specific characteristics. The wormhole detection methods are classified into three categories, namely: (a) Time-based approaches, (b) Location-based approaches, and (c) Neighborhood- based approaches.

### 1.1 Time-based approaches

The first type of wormhole detection methods is the Time-based approach in which the anomaly is detected based on the time mismatch of the forwarding packets [42]. In [13], Yih-Chun et al. proposed temporal leash method based on tightly synchronized clocks to restrict the maximum transmission time from source to destination. Using this method in sensor networks requires applying time synchronization level that is impractical [43]. Another method is designed by Karlsson et al. [44] to detect the wormhole attack in MANET based on Traversal Time and Hop Count Analysis (TTHCA). Hon Sun et al. [45] considered the rate of delay per hop and introduced a method that is named DELPHI. By comparing the normal path and a path with wormhole attack with same hop, the authors found that when the wormhole happens, the rate of delay will be more than the normal path. Qazi et al. [46] enhanced the security of dynamic source routing (DSR) protocol against wormhole attacks based on calculation of round trip time (RTT).

### 1.2 Location-based approaches

The second group of wormhole detection approaches is known as the location-based approaches. The core idea behind such a method is to use the location information of the nodes to identify the malicious nodes [47]. Yih-Chun et al. [13] was the first to introduce a location-based approach, namely the Geographic Leashes in which all of the nodes know their location and use a loosely synchronized clock to achieve global serialization. When the packet is sent to the destination, each node attaches the transmission time and its location to packet. After the packet is successfully received by the next node, firstly, the distance to sender and the traversal time are calculated to determine the wormhole attack. The work of Lazos et al. [48] is an attempt to detect wormhole attack in ad hoc networks on the basis of establishing Local Broadcast Keys (LBK) for nodes, that is called graph theoretic approach (GTA). This method also needs to use a special localization equipment and GPS. However, the main drawback of such a model is that it is not readily applicable to mobile networks. In [49], the authors introduced a method to detect the wormhole attack in MANETs based on the distance verification and using the received signal strength (RSS) and statistical hypothesis testing. The main idea of the distance verification is to verify the computed distance using a RRS measured distance to the sender.

### 1.3 Neighborhood-based approaches

In the neighborhood-based approaches, the wormhole attack is detected by analyzing the characteristics of the neighbor nodes. The localized encryption and authentication protocol (LEAP+) is a detection approach implemented by Zhu et al. [50] based on clustering and defining four types of key for each sensor node: (a) a pairwise key shared with another sensor node, (b) an individual key shared with the base station, (c) a cluster key shared with multiple neighboring nodes, and (d) a group key that is shared by all of the nodes in the network. The drawback of this model is that it can only be applicable for static or immobile sensor networks and is not readily applicable to the mobile networks. The multipath hop-count analysis (MHA) is implemented based on analyzing the hops to avoid wormhole attack in MANETs [43]. The MHA method consists of three steps: (a) calculating the hop-count values of all routes to the destination, (b) selecting secure set of routes for data transmission, and (c) broadcasting the packet through the safe routes. Generally, it helps when the wormhole attacks occur in a way that the number of hops will be lesser than normal situation. However, this method is impractical when the malicious nodes are able to create the virtual hops to deceive the detection algorithm.

Xu et al. [51] proposed a wormhole geographic distributed detection (WGDD) approach to detect a wormhole by using a hop count technique, reconstructing local maps in each node, and then using a “diameter” feature to detect abnormalities caused by wormholes. Another mechanism is end-to-end detection of wormhole attack in wireless ad-hoc networks (EDWA) [52], which is used to detect wormhole attacks in an ad-hoc routing protocol based on hop-count scenario. EDWA has two steps: (1) detection of a wormhole by estimating shortest path, and (2) identifying the malicious nodes based on the shortest path. After that the source node compares the hop-count which is retrieved from the Route Reply (*h*_*r*_) with the number of hops within the shortest path to the destination (*h*_*e*_). The wormhole attack has occurred if and only if *h*_*r*_ ≤ *h*_*e*_. However, this model also has several assumptions which have to be considered like using Global Positioning System (GPS) and using TESLA for authentication. Song and Li [53] designed another mechanism in multi-path routing wireless ad-hoc networks based on Statistical Analysis of the Multi-path (SAM). The main idea behind this method is to monitor the statistics of the route discovery that dramatically changes under wormhole attack.

Wormhole attack is able to effect geographic routing protocols. However, there is a little work that focused on detecting wormhole on the basis of geographic routing protocols such as RRS and ANS. Poornima and Bindhu [54] proposed two different methods for dynamic and static wormholes. Reverse Routing Scheme (RRS) is the first technique that attempts to recognize static wormhole attack by means of Hop-Count mechanism. The author of RRS method has indicated that this method is related to choosing a proper threshold. However, selecting a threshold is not always easy. Furthermore, this method cannot identify a high number of malicious nodes. Authentication of Nodes Scheme (ANS) [54] is the next method that uses digital signature to prevent the wormhole attacks. Once a packet is directed to the destination, each node is responsible to insert its digital signature in the forwarding packet. Since the malicious nodes are not able to sign the transmitted packet without the key, the author claims, the destination node can find them easily. However, the performance of this method is based on digital signature (RSA). It is assumed that just the trusted nodes have a reliable signature. If the adversary is able to break RSA, then there are no obstacles to create wormholes. Furthermore, the computational cost involved in signing every packet is very high. On the other hand, the verification of intermediates nodes is determined just in the destination node. This process has several consequences for the network functionality

## 2 Proposed Wormhole Detection Method

This section presents the proposed scheme of wormhole attack detection in geographical routing protocol (DWGRP). One of the main characteristics of our scheme, which makes it different from the previous approaches, is to detect and eliminate the malicious nodes before the packet is sent to the destination. Furthermore, if the adversary breaks the trust level between two adjacent nodes that is generated using an updated version of the pairwise key [23], the certification of this path is denied in the destination. The DWGRP approach consists of three main steps, as follows:

(a) Deploying nodes: generating the new pairwise key to construct neighborhood tables.
(b) Intermediate step: identifying trust neighbors and detecting malicious nodes with respect to the secure shared keys.
(c) Destination step: identifying untrusted packets upon receiving them at the destination.

**Figure 5:**
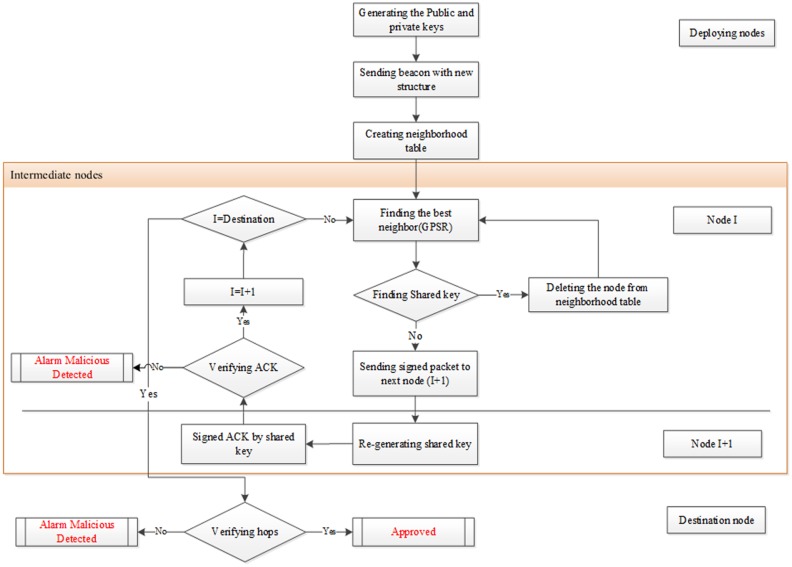
Wormhole detection algorithm.

Fig. 5 displays the flowchart of the proposed wormhole detection method (DWGRP) [55].The rest of this section explains in detail the wormhole attack proposed detection method in geographical routing protocol.

### 2.1 Key Management phase

Each sensor node requires a pair of public and private keys to communicate with the other nodes through the secure channel. We propose a new pairwise distributed key secure based on one-way hash function to generate the public and private key for sensor node, as follows:

#### 2.1.1 Public key generation

The key pool (*KP*) is a matrix with size (*y* + 1) × *N* where *N* is number of sensor nodes and *y* is desired level of trust or the number of potential private keys. We construct this matrix by using a secure one-way hash function *x*_*i*_ = *Hash*(*k*, *N*), where *k* is assumed the smallest prime number larger than 2^64^. Then, a key pool can be designed as follows:

$$KP={\left[\begin{array}{ccccc} 1 & 1 & 1 & \ldots & 1\\ x_{i} & x_{i}{}^{2} & x_{i}{}^{3} & \ldots & x_{i}{}^{N}\\ x_{i}{}^{2} & {\left(x_{i}{}^{2}\right)}^{2} & {\left(x_{i}{}^{2}\right)}^{3} & \ldots & {\left(x_{i}{}^{2}\right)}^{N}\\ \; & \ddots & \; & \vdots & \;\\ x_{i}{}^{y} & {\left(x_{i}{}^{y}\right)}^{2} & {\left(x_{i}{}^{y}\right)}^{3} & \ldots & {\left(x_{i}{}^{y}\right)}^{N} \end{array}\right]}_{(y+1)\times N}$$ (1)

Since the sensor nodes have constrained resources, each node *i* only need to store the corresponding seed *x*_*i*_ to generate its column as a public key.

#### 2.1.2 Private Key generation

After generating the key pool, the private key of the sensor nodes needs to be computed. Firstly, *y* symmetric matrixes with size (*y* + 1) × (*y* + 1) are calculated by using a secure one-way hash function, as follows:

$$Pr_{i}={\left[\begin{array}{ccccc} 1 & x_{i} & x_{i}^{2} & \ldots & x_{i}^{y}\\ x_{i} & x^{2} & {\left(x_{i}^{2}\right)}^{2} & \ldots & {\left(x_{i}^{y}\right)}^{2}\\ x_{i}^{2} & {\left(x_{i}^{2}\right)}^{2} & {\left(x_{i}^{2}\right)}^{3} & \ldots & {\left(x_{i}^{y}\right)}^{3}\\ \; & \ddots & \; & \vdots & \;\\ x_{i}^{y} & {\left(x_{i}^{y}\right)}^{2} & {\left(x_{i}^{y}\right)}^{3} & \ldots & {\left(x_{i}^{y}\right)}^{y} \end{array}\right]}_{\left(y+1\right)\times \left(y+1\right)}$$ (2)

where *x*_*i*_ = *Hash*_*i*−1_(*k*,*N*), and 1 ≤ *i* ≤ *y*. Then, the private key matrixes of sensor nodes with size (*y* + 1) × *N* are computed by:

$$P_{i}\;=\;{\left(Pr_{i}\;*\;KP\right)}^{T}$$ (3)

As a result, *y* potential private keys are computed for each node (*i*) that consists of the corresponding row (row_*i*_) from matrixes *P*_1_ *P*_2_…*P*_*y*_. Due to the resource restriction of sensor nodes, we select *α* private keys for each node (2 ≤ *α* ≤ *y*) randomly among of these *y* potential private keys.

### 2.2 Creating neighborhood table

**Figure 6:**
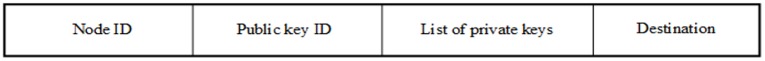
Structure of beacon Packet.

After generating the private keys, each node sends a beacon packet which includes ID, location and destination, to find its neighbor nodes and update its neighborhood table. In order to find malicious nodes, we modify the beacon packets and the neighborhood tables structure of the sensor nodes by inserting the list of the private key matrixes (*P*_*i*_) into the beacon packet. In other words, the new beacon packet includes Node ID, Location, ID of Public key (*KP*), a list of matrix numbers of private keys and destination. The structure of beacon is illustrated in Fig. 6.

**Figure 7:**
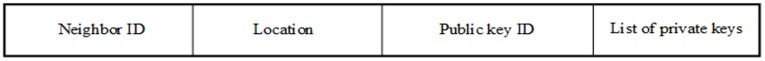
Neighborhood table.

When the beacon packet is received by the neighbor nodes, they update their neighborhood table. If the node ID is found in the table, its location, the public key and list of private keys are updated otherwise one row is added to the neighboring table of the node. Fig. 7 shows the structure of neighborhood table.

### 2.3 Intermediate step

Regarding the GPSR protocol, the best neighbor node is the nearest node to the destination. We modify this rule in GPSR and add a restriction for selecting the best neighbors based on the list of private keys. In other words, when node *A* finds the best neighbor *B* from its neighborhood table, the list of private keys of node *A* needs to be compared with the list of private keys of node *B*. Then, node *B* is selected as a next node if and only if at least one private key of node *A* and *B* is selected from the same matrix. If they have no similar matrices, node *B* is eliminated from the neighborhood table and this phase repeats again to find the best neighborhoods.

$$B=\;best\;neighbour\;of\;A\;↔\;\exists \;\;P_{i}|Pr_{B}\subset P_{i}and\;Pr_{A}\subset P_{i}$$ (4)

where the list of private keys of node *B* is *Pr*_*B*_ and *Pr*_*A*_ is the list of private keys of node *A*.

#### 2.3.1 Shared key generation phase

When the best neighbor is selected, the node needs a shared key to communicate with its neighbors securely. The shared key is created for each pair of two nodes that have a private key from the same matrix Pi. For example, if nodes *i* and *j* have a key form matrix *P*_1_, the shared key can be calculated by:

$$P_{1}*\;K\text{P}={\left(Pr_{1}\;\text{*}\;\text{K}P\right)}^{T}*K\text{P}=KP^{T}*Pr_{1}^{T}*KP$$ (5)

We have mentioned it earlier that *Pr* is symmetric matrix, then $Pr_{1}^{T}=Pr_{1}$. Therefore, *P*_1_ * *kP* is equal to:

$$\begin{array}{l} P_{1}*KP=KP^{T}*Pr_{1}*KP=KP^{T}*(Pr_{1}*KP)\\ \Rightarrow P_{1}*KP={\left({\left(Pr_{1}*KP\right)}^{T}*KP\right)}^{T} \end{array}$$ (6)

Then, the result of *P*_1_ * *kP* is a symmetric matrix with size *N* × *N* in which *SK*_*ij*_ = *SK*_*ji*_ is the shared key between nodes *i* and *j*. After generating the shared secret key, the packet and the selected index are sent to the best neighbor by using the shared key. It is important to mention that we assume our network is completely dense and each node has at least one neighbor with the same specification.

#### 2.3.2 Verification phase

When the packet is received by the best neighbor (for example node *B*), this node extracts the index from the packet and regenerate the shared key. Then, node *B* creates an ACK message, signs it by using a shared key, and sends it to node *A*. If the ACK message is verified by node *A*, node *B* is a trusted node, otherwise node *B* is a malicious node. Therefore, Node *A* generates an alarm message and broadcast to the network that the node for eliminating the malicious node from their neighborhood tables.

### 2.4 Destination Step

When a packet is received by the destination node, the probability of wormhole attack happening is checked based on the distance between source node to the destination node and the number of hops from source to destination. A necessary condition for detecting wormhole in destination node is:

$$\sqrt{{\left(x_{d}-x_{s}\right)}^{2}+{\left(y_{d}-y_{s}\right)}^{2}}>R*h$$ (7)

where the location of the source node is (*x*_*s*_, *y*_*s*_), (*x*_*d*_, *y*_*d*_) is the location of the destination, *R* is the radio range of nodes, number of hops from source to destination is shown by *h*. When the wormhole is detected, a request packet is sent to source node in order to send a packet again from another path.

## 3 Evaluation

We conduct the mathematical modelling and extensive simulations under various situations to evaluate the effectiveness of our wormhole detection approach. We evaluate the probability of miss detection and successful wormholes detection by altering the node density and the number and type of wormholes inside the network.

### 3.1 Miss detection probability analysis

Miss detection probability is a crucial metric to evaluate wormhole detection methods. According to key distribution of this method, there are *y* potential private keys for *N* sensor nodes in the network and each sensor node randomly selects *α* private keys (*α* ≤ *y*). Therefore, the probability of a specific key belonging to one node is equal to $\left\lfloor \frac{\alpha }{y}\right\rfloor$ while the malicious nodes do not have a private key. If the adversary is able to compromise *x* nodes, the probability that just *m* nodes from *x* nodes (*m* ≤ *x* ≤ *N*) contain the specific key (*K*_*i*_) from *y* potential private keys is computed by:

$$P(k_{i})=\left(\begin{array}{c} x\\ m \end{array}\right){\left(\frac{\alpha }{y}\right)}^{m}{\left(1-\frac{\alpha }{y}\right)}^{x-m}$$ (8)

Therefore, the probability of breaking one link by an adversary is calculated in below formula:

$$P(l)=\sum_{m=1}^{x}\left(\begin{array}{c} x\\ m \end{array}\right){\left(\frac{\alpha }{y}\right)}^{m}{\left(1-\frac{\alpha }{y}\right)}^{x-m}$$ (9)

Where *x* is the number of compromised nodes, numbers of selected nodes are shown by *m*, *y* is the number of potential private keys and *α* is equal to the number of private keys for each node.

**Figure 8:**
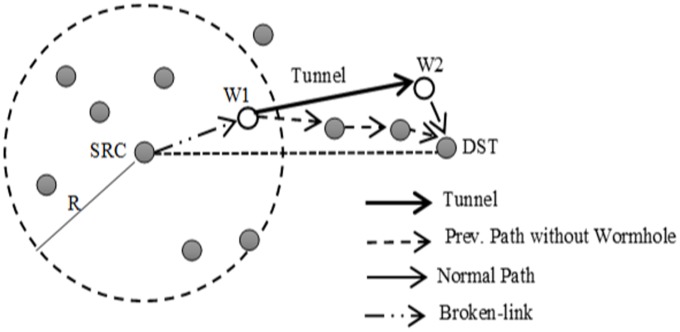
Creating wormhole by breaking two link in geographic routing protocols.

To create a wormhole in geographic routing protocol necessitates two or more malicious nodes to receive packets at one point of the network and forward those packets to another location by a wireless or wired tunnel. Therefore, as shown in Fig. 8, the adversary needs to break more than 1 link to create the wormhole attack in the network.

If there are *h* hops from source node to destination, the adversary needs to break *j* link (*j* < *h*) to create wormhole attack. Then, the probability of creating wormholes by the attacker is:

$$\begin{array}{c} P(Wormhole)\;=\;\left(\begin{array}{c} j\\ h \end{array}\right)P{\left(l\right)}^{j}{\left(1-P(l)\right)}^{h-j}\\ =\left(\begin{array}{c} j\\ h \end{array}\right).{\left(\sum_{m=1}^{x}\left(\begin{array}{c} x\\ m \end{array}\right){\left(\frac{\alpha }{y}\right)}^{m}{\left(1-\frac{\alpha }{y}\right)}^{x-m}\right)}^{j}.{\left(1-\sum_{m=1}^{x}\left(\begin{array}{c} x\\ m \end{array}\right){\left(\frac{\alpha }{y}\right)}^{m}{\left(1-\frac{\alpha }{y}\right)}^{x-m}\right)}^{h-j} \end{array}$$ (10)

where *h* = the number of hops, *j* = the number of broken links, *x* = the number of compromised nods, *α* indicates the number of private keys for each node and *y* is the number of potential private keys.

**Figure 9:**
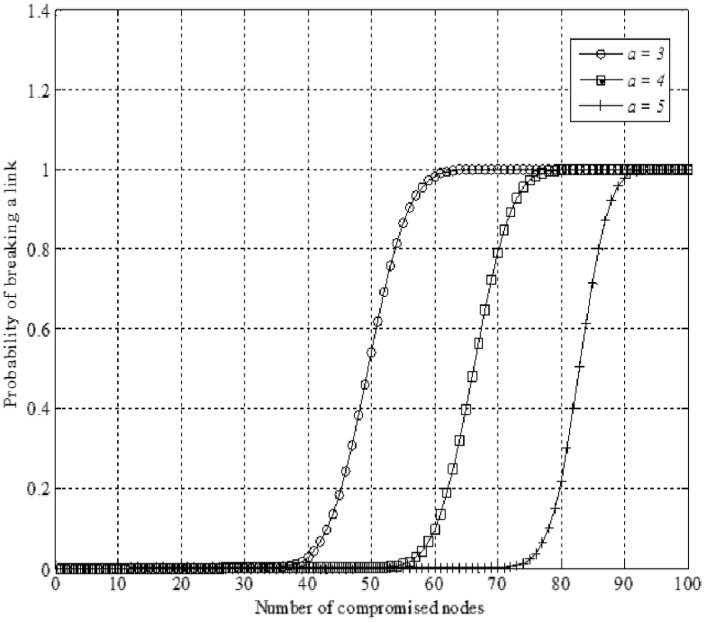
Probability of breaking a link when *y* = 6 and (*α* = 3, 4, or 5).

Fig. 9 shows the probability of a broken link where the number of potential private keys is 6 (*y* = 6), the number of private keys is 3, 4, or 5 (*α* = 3, 4, or 5), the maximum number of compromised nodes is 100 (x = 100) and the number of hops from source to destination node is equal to 10 (*h* = 10). It can be seen that when the amount of compromised nodes are less than 55, the attacker cannot break the link, but with the increase of the number of compromised nodes, the probability of broken link is increasing too till the number of compromised node is equal to 70 and the attacker can break the link. Fig. 9 also shows that when the number of compromised nodes is less than 55 or more than 65, the adversary cannot create the wormhole attack otherwise the maximum rate of miss detection wormhole is about 0.3 when the number of compromised nodes is from 55 to 65 (S1 Table).

**Figure 10:**
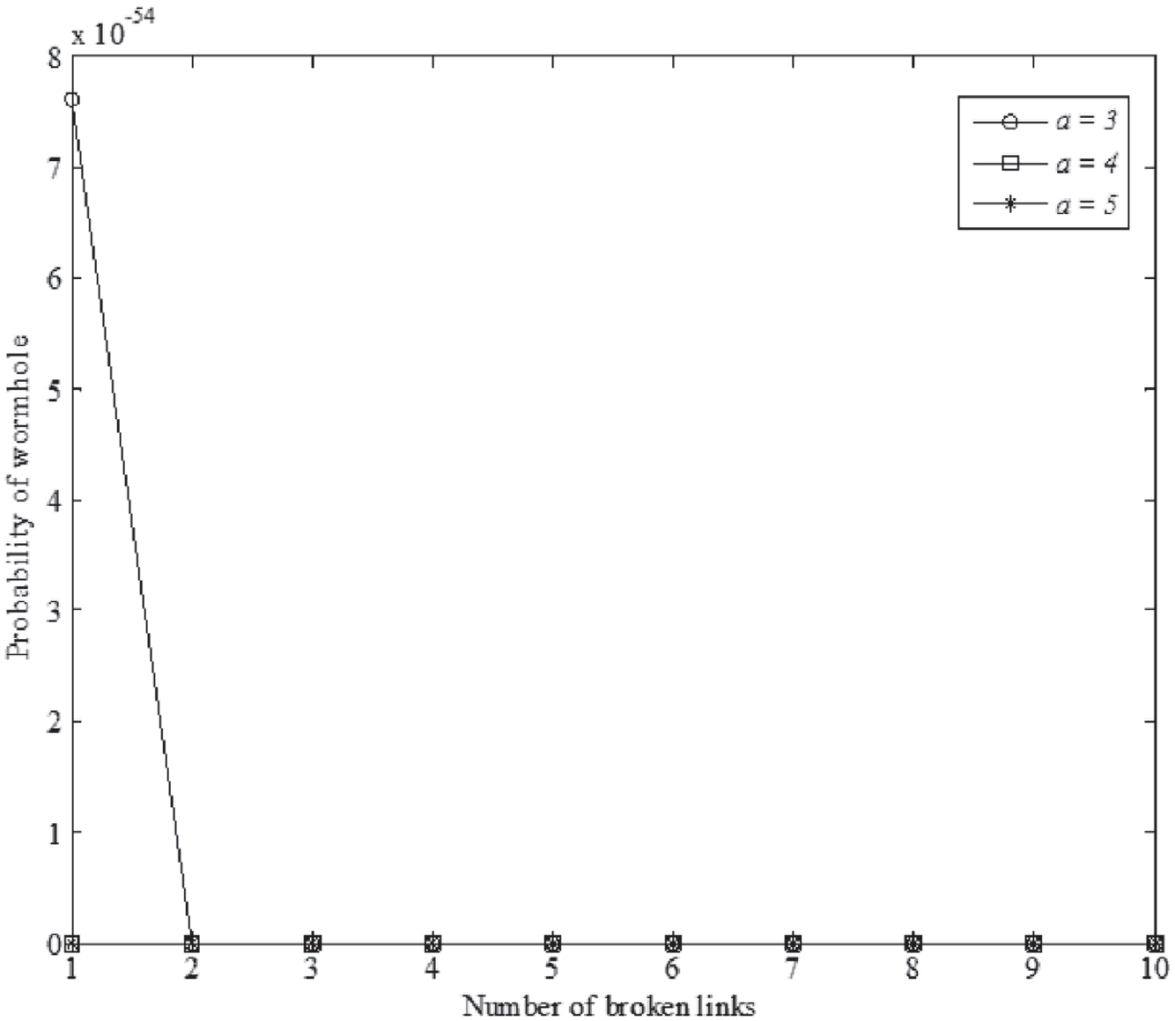
Probability of miss detection *y* = 6 and (*α* = 3, 4, or 5).

In Fig. 10, the comparison of the probability of miss detection is illustrated when the number of private keys is 3, 4, or 5 (*α* = 3, 4, or 5) and the number of hops from source to destination node is equal to 10 (*h* = 10). As it is shown, our method is able to detect almost all wormhole attacks (S2 Table).

### 3.2 Simulation Setup

We use simulator NS-2 to evaluate the performance of our scheme for detecting wormhole attack in geographic routing protocols. We deploy 200 nodes randomly in a square area of size 1000 * 1000 *m* ^2^ square area with multiple holes inside. The transmission range of each node is set to 150 *m*. The number of malicious nodes is considered between 2 to 10 nodes, which can change their location to create the wormhole attack. We implement the GPSR routing protocol and then improve the structure of beacon and neighborhood table based on our method. Each node broadcasts the beacon packets periodically with a nominal interval of 0.3 seconds to update its neighborhood table. We present our results after averaging of 100 simulation runs. All simulation parameters are shown in the Table 1.

**Table 1:** Simulation Parameters.

**Simulation Parameters**	**Value**
Routing Protocol	GPSR
Number of Nodes	200
Transmission range	150 *m*
Malicious nodes	2–10
Packet Size(bytes)	512
Traffic Type	CBR
Paused time	50 sec
Movement Model	Random Way Point
Number of wormhole	1–5
Simulation area(*m* ^2^)	1000*1000

**Figure 11:**
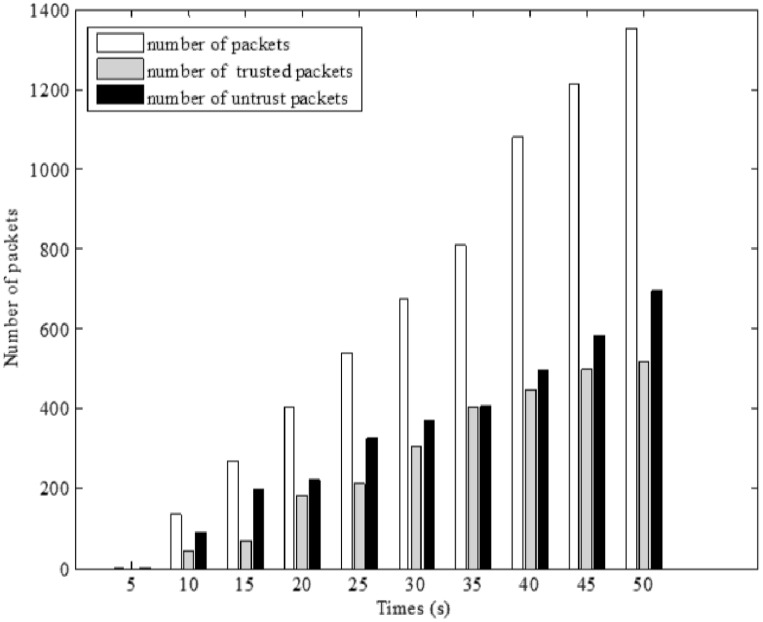
The effect of wormhole attack on geographic routing protocols.

Before simulating our method, the impact of wormhole attacks on geographic routing protocols is illustrated in Fig. 11. We transfer 1250 packets within 20 hops and monitor the network to find the number of packets that are sent through the malicious nodes, are called untrusted packets. As it is clear, the wormhole attack is capable of transferring nearly 60% of the packets. Since the malicious nodes are able to change or drop the packets, it is necessary to protect this network against wormhole attack (S3 Table).

#### 3.2.1 Wormhole detection rate

**Figure 12:**
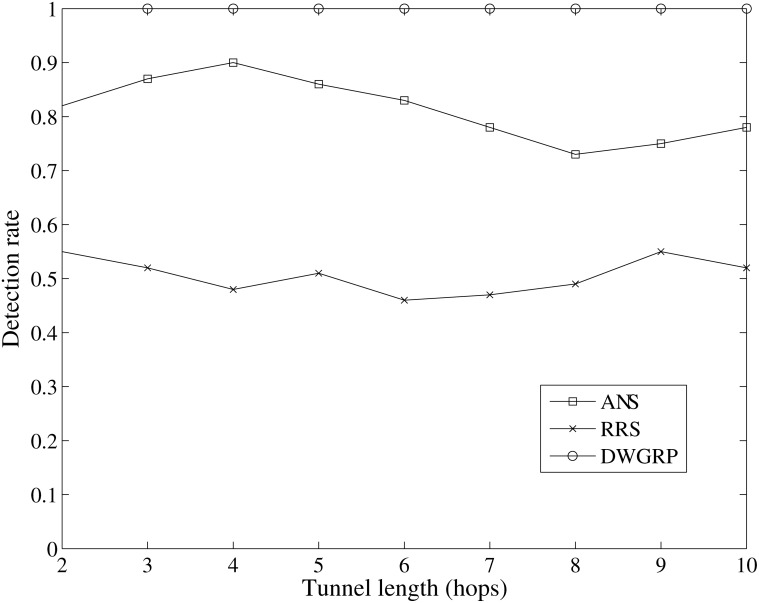
Wormhole detection rate against different tunnel length.

The wormhole detection rate is defined as the ratio of the number of detected wormhole over the total number of attacks by the adversaries in the network. Fig. 12 plots the wormhole detection rate versus the tunnel length. We randomly place one wormhole in the network in each run. It can be seen that our scheme (DWGRP) is able to detect all wormhole attacks (100%). However, the rate of wormhole detection in ANS method is approximately 80 percent. RRS method has the minimum rate of wormhole detection which is about 50 percent when the length of the tunnel reaches to 10. Generally, the performance of the DWGRP method to detect the wormhole attack is satisfactory and better than the other methods (S4 Table).

**Figure 13:**
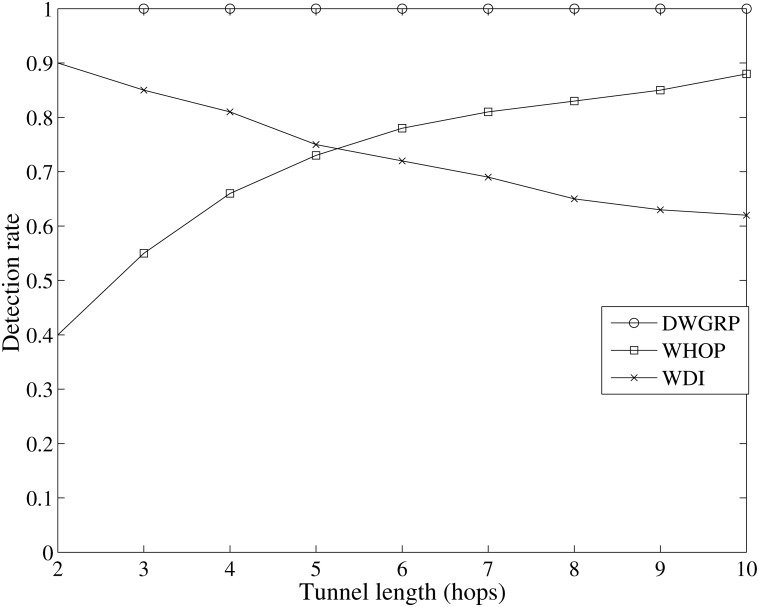
Wormhole detection rate versus tunnel length in WDI, WHOP and DWGRP.

Then, we compare the rate of wormhole detection for our scheme (DWGRP) with WHOP [56] and WDI [57]. We simulate the WHOP and WDI methods on GPSR protocols to compare their performance. As it is shown in Fig. 13, the wormhole detection rate in WDI method has a slightly downward trend in which by increasing the tunnel length, the detection rate decreases from 90% to 70%. In WHOP scheme, the detection rate rises up to nearly 85% when the number of hops approaches 10. Since the DWGRP scheme is able to detect 100% of wormhole attacks, the performance of our scheme is better than WHOP and WDI in geographic routing protocols (S5 Table).

#### 3.2.2 Resend packet rate

**Figure 14:**
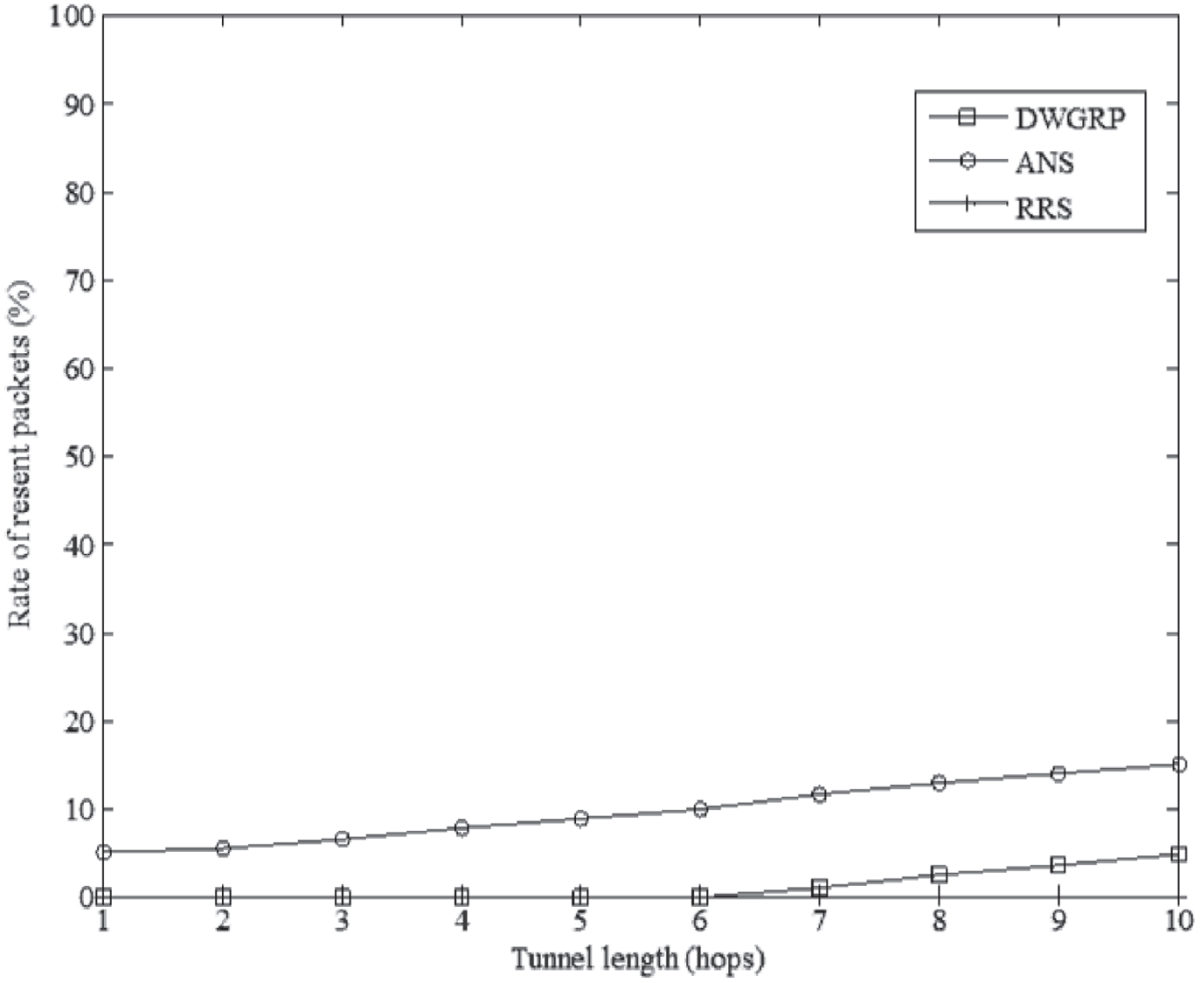
The rate of re-send packets against different tunnel length.

If a wormhole attack is detected by the sink in our scheme, this packet will be eliminated and the packet will be re-sent. The ratio of the number of re-send packets over the total number of packets from source to destination is defined as the rate of re-send packets. Therefore, by increasing the rate of re-send packets, the rate of wasted energy is increased in the network. Fig. 14 shows the rate of re-send packets against different tunnel lengths for DWGRP, ANS and RRS. The rate of re-send packets in our scheme is zero when the tunnel length is less than 7. When the tunnel length reaches to 10, less than 5 percent of packets need to be re-sent in DWGRP because the attacker cannot break the link in the network. However, approximately 15 percent of packets which arrive to the sink will be re-sent in ANS method when the tunnel length is equal to 10. In the RRS scheme, the sink node cannot detect the wormhole attack and request re-sending packets. Therefore, this rate is zero for RRS (S6 Table).

## 4 Conclusion

Wormhole attack is recognized as a severe threat to wireless sensor networks and geographic routing protocols. Detection of wormhole attack is difficult because such attacks appear in various modes. In this paper we categorize the wormhole attacks based on their characteristics and impact on different routing protocols. Then, we present a novel detection method of wormhole attacks in geographic routing protocols by improving the pairwise key pre-distribution scheme based on the beacon packets. Our scheme has the capability to detect the malicious nodes before receiving the message. The proposed scheme does not need any special hardware devices and additional assumptions, such as network synchronization, special guard nodes, or unit disk communication model. Simulation results and analytical modeling show that DWGRP approach achieves superior performance and applicability with the minimum restrictions compared with the related works in geographic routing protocols or wireless sensor networks. For the future work, we intend to improve this method by modifying the pairwise key pre-distribution scheme to detect all malicious nodes. We will also improve our method to prevent the Sybil attack.

## Supporting Information

S1 Table Probability of breaking in different scenarios. (XLSX)

S2 Table Probability of miss detection in different scenarios. (XLSX)

S3 Table The effect of wormhole attack on geographic routing protocols. (XLSX)

S4 Table Comparison of wormhole detection rate in ANS, RRS, and the proposed method based on length of tunnel. (XLSX)

S5 Table Comparison of wormhole detection rate in WDI, WHOP and the proposed method based on length of tunnel. (XLSX)

S6 Table Comparison of re-send packets in traditional and the proposed method based on length of tunnel. (XLSX)
